# Acute Toxicity and Genotoxicity of cMoL, a Lectin From *Moringa oleifera* Seeds With Antifungal Activity Against *Cryptococcus* Strains

**DOI:** 10.1002/jat.4922

**Published:** 2025-09-09

**Authors:** Matheus Cavalcanti de Barros, Sávia Soraia Santana da Silva, Eduarda Pinto Gagliano, Alícia Natalie Silva dos Santos, Leydianne Leite de Siqueira Patriota, Gustavo Ramos Salles Ferreira, Pollyanna Michelle da Silva, Simone da Paz Leôncio Alves, Julliano Matheus de Lima Maux, Jacinto da Costa Silva Neto, Fernanda Rafaelly Nascimento de Santana, Fabiana Aparecida Cavalcante Silva, Marilene Henning Vainstein, Luana Cassandra Breitenbach Barroso Coelho, Thâmarah de Albuquerque Lima, Thiago Henrique Napoleão, Patrícia Maria Guedes Paiva

**Affiliations:** ^1^ Departamento de Bioquímica Universidade Federal de Pernambuco Recife Pernambuco Brazil; ^2^ Departamento de Farmácia Universidade Federal de Pernambuco Recife Pernambuco Brazil; ^3^ Departamento de Histologia e Embriologia Universidade Federal de Pernambuco Recife Pernambuco Brazil; ^4^ Centro de Tecnologias Estratégicas do Nordeste Recife Pernambuco Brazil; ^5^ Departamento de Biologia Molecular e Biotecnologia, Instituto de Biociências Universidade Federal do Rio Grande do Sul Porto Alegre Brazil

**Keywords:** antifungal protein, antimicrobial lectin, cryptococcosis, toxicology

## Abstract

Coagulant *Moringa oleifera* lectin (cMoL) is one of the compounds involved in the application of *M. oleifera* seeds for traditional water treatment methods. The present study highlights the new biotechnological potential of cMoL lectin as an antifungal agent against *Cryptococcus neoformans* B3501 and H99 and *Cryptococcus gattii* R265 strains. Acute toxicity and genotoxicity were assessed to provide information on security utilization. The antifungal activity was evaluated using the broth microdilution test, flow cytometry, antibiofilm activity, and synergistic effects with fluconazole. Acute toxicity was determined by administering a single dose of cMoL (200 mg/kg) to female Swiss albino mice and examining the following parameters: water and feed consumption, weight variation, blood hematological and biochemical parameters, serum cytokine levels, and histopathological analyses. Genotoxicity was assessed using comet assay and micronucleus test. cMoL inhibited the growth of all tested isolates, with a minimal inhibitory concentration of 7.5 μg/mL. Flow cytometry data showed that cMoL (7.5 μg/mL) significantly decreased cell viability by promoting necrosis. cMoL was not found to be an antibiofilm agent against *Cryptococcus* species; however, it acted synergistically with fluconazole against *C. neoformans* H99. cMoL (200 mg/kg) did not cause animal death or toxicity. No hematological, biochemical, histopathological, or genotoxic effects were observed. These results encourage the exploration of cMoL as a natural and safe antifungal agent against *Cryptococcus*.

## Abstract 2

The coagulant *Moringa oleifera* lectin (cMoL) demonstrated antifungal activity against *Cryptococcus neoformans* and *Cryptococcus gattii*, with synergistic effects observed with fluconazole against *C. neoformans* H99. cMoL significantly reduced fungal viability by inducing necrosis and showed no antibiofilm activity. Acute toxicity and genotoxicity assessments in mice revealed no adverse effects at 200 mg/kg. These results support the safe and promising biotechnological application of cMoL as a natural antifungal agent.

## 1 Introduction

*Moringa oleifera* seeds are used to treat water for human consumption because of the presence of coagulant organic polyelectrolytes and proteins, such as lectins (Ndabigengesere et al. 1995; J. T. A. Oliveira et al. 1999; Okuda et al. 2001; R. Ferreira et al. 2011). Lectins are proteins that are capable of establishing reversible and specific bonds with carbohydrates and glycoconjugates (Goldstein et al. 1980; Lossio et al. 2017). The carbohydrate‐recognition ability of lectins is related to a series of biological activities, including anticancer, antimicrobial, antiparasitic, insecticidal, and immunomodulatory activities (Ramos et al. 2019; Napoleão et al. 2011; Aranda‐Souza et al. 2018; W. F. Oliveira et al. 2020; A. J. C. A. Santos, Barros, et al. 2020; J. D. F. Silva et al. 2019). In addition to these biological applications, lectins may function as antinutritional factors in humans, eliciting symptoms such as vomiting, diarrhea, and abdominal pain (S. He et al. 2018). In vivo toxicological studies have demonstrated the adverse effects associated with lectins, including alterations in hepatic enzyme levels, changes in leukocyte counts, genotoxicity, and mortality (Chaturvedi et al. 2015; Jin et al. 2014; J. S. Brito et al. 2023) The toxic effects of lectins are influenced by their dosage, frequency of exposure, and route of administration (Jin et al. 2014; Kuku et al. 2012).

The coagulant *M. oleifera* lectin (cMoL) is a compound associated with the application of *M. oleifera* seeds for water treatment. cMoL is a cationic protein (pI = 11.67) with a molecular weight of 26.5 kDa and is composed of 101 amino acids (Luz et al. 2013). cMoL reduces the turbidity of a clay suspension, similar to aluminum sulfate, a coagulant used for water treatment (A. F. Santos et al. 2009). The immobilization of cMoL in a chromatographic column efficiently removes humic acid, an organic pollutant, from water (A. F. Santos et al. 2011). cMoL exhibits insecticidal activity against *Anagasta kuehniella*, an economically significant pest that feeds on stored products, and inhibits superoxide dismutase activity in the larvae of *Aedes aegypti* resistant to organophosphates (Agra‐Neto et al. 2014; C. F. R. Oliveira et al. 2011). cMoL also interferes with the motility of *Haemonchus contortus* larvae, a hematophagous parasite that damages ruminant production worldwide (Medeiros et al. 2020). Additionally, cMoL causes cell death in murine melanoma cells (B16‐F10) by inducing necrosis and apoptosis at noncytotoxic concentrations (Luz et al. 2017). Finally, cMoL also exhibits anti‐inflammatory activity in lipopolysaccharide‐stimulated murine macrophages, regulating the production of nitric oxide, TNF‐α, and IL‐1β (Araújo et al. 2013). In vitro toxicity of cMoL has been previously evaluated, and the results showed low toxicity to human fibroblasts (Luz et al. 2017) but potential toxicity to peripheral blood mononuclear cells (PBMCs) (Araújo et al. 2013). Therefore, an in vivo toxicity assessment of cMoL is necessary considering its potential biotechnological applications.

Lectins have been recognized as fungicidal agents because of their capacity to interact with glycans located on the cell surface, resulting in pore formation, alterations in cellular permeability, and the disruption of cell wall synthesis (Coelho et al. 2018). Scientific publications have reported antifungal activities of plant lectins against species of *Cryptococcus* (G. R. S. Ferreira et al. 2023; Jones et al. 2017; L. M. M. Santos et al. 2024). *Cryptococcus* species cause cryptococcosis, a systemic disease that affects immunocompetent or immunocompromised hosts, and the species *Cryptococcus neoformans* and *Cryptococcus gattii* are the most clinically relevant (May et al. 2016; Perfect and Bicanic 2015; Surawut et al. 2019). The route of infection involves the airways; however, these fungi also affect organs other than the lungs, such as the brain, causing cryptococcal meningitis (Krysan 2015; Li and Mody 2010). Cryptococcal infections are challenging to treat because of the unique characteristics of the *Cryptococcus* genus, such as genomic plasticity and physiological adaptability, which proffer resilience to antifungal assault (Iyer et al. 2021). The virulence factors of *Cryptococcus* include thermotolerance, the presence of a capsule, melanin synthesis, and biofilm formation (Gibson and Johnston 2015). The high toxicity of currently available drugs and antifungal resistance of *Cryptococcus* highlight the need to develop new treatments for cryptococcosis (Perfect et al. 2010). *C. neoformans* is within the critical group with the highest importance for public health on the list of fungal priority pathogens (World Health Organization 2022).

The previously reported in vivo and in vitro bioactivities of cMoL, along with the previously reported antifungal potential of plant lectins, and the need to develop new strategies for the treatment of cryptococcosis inspired the evaluation of the antifungal potential of lectins against *Cryptococcus* species. In addition, acute toxicity and genotoxicity studies in mice were conducted to evaluate the safety of cMoL to guarantee secure biotechnological applications of this protein.

## 2 Material and Methods

### 2.1 Purification of Coagulant *M. oleifera* Seeds Lectin

*M. oleifera* seeds were collected from the city of Arara, Paraíba, Brazil, with authorization (No. 72.024) from the *Instituto Chico Mendes de Conservação da Biodiversidade* (Brazilian Ministry of Environment). cMoL was purified as previously described by A. F. Santos et al. (2009). The first step of lectin isolation was to obtain a saline extract from the seeds through homogenization of seed powder (10 g) with 0.15‐M NaCl (100 mL) for 6 h at 28°C under agitation with a magnetic stirrer. Thereafter, the extract was filtered through gauze and treated with saturated (60%) ammonium sulfate for 4 h with subsequent rest overnight at 7°C. The precipitate corresponding to the protein‐enriched fraction was collected and subjected to dialysis (3500‐Da cut‐off membrane) for 4 h (three changes with distilled water and one with 0.15‐M NaCl). Subsequently, the fraction was applied to a guar gum column (7.5 × 1.5 cm) equilibrated with 0.15‐M NaCl. Adsorbed cMoL was eluted with 1.0‐M NaCl, which was monitored by measuring the absorbance at 280 nm. Isolated cMoL was dialyzed against distilled water, lyophilized, and stored in a freezer until testing. The carbohydrate‐binding ability of cMoL was assessed using the hemagglutinating activity test (Paiva and Coelho 1992) with rabbit erythrocytes (authorized by the Ethics Committee on the Use of Animals at Universidade Federal de Pernambuco [UFPE], Process No. 23076.033782/2015‐70). Protein concentration was determined using protein absorbance at 280 nm, molecular weight, and the extinction coefficient of cMoL (3520 M^−1^ cm^−1^) (AAT Bioquest Inc 2025).

### 2.2 Antifungal Activity Assays

#### 2.2.1 Microorganisms

*C. neoformans* B3501 (Serotype D), *C. neoformans* H99 (Serotype A), and *C. gattii* R265 (Serotype B) were obtained from the Culture Collection of the *Laboratório de Fungos de Importância Médica e Biotecnológica* of the Universidade Federal do Rio Grande do Sul (Porto Alegre, Brazil). Stock cultures were maintained at −80°C in sterile nonfat milk powder with 10% glycerol (v/v). The fungi were cultivated in Sabouraud dextrose agar (SDA) for 48 h at 30°C. The colonies were then removed and added to sterile 0.15‐M NaCl to obtain suspensions at 3 × 10^6^ colony‐forming units (CFU) per milliliter through spectrophotometric determination at 600 nm.

#### 2.2.2 Determination of Minimal Inhibitory Concentration

First, cMoL was diluted in sterile (autoclaved) saline solution (0.15‐M NaCl) and filtered using a 0.22‐μm pore size filter. An aliquot of the filtered protein was used to determine the protein concentration using the previously described extinction coefficient. After sample preparation, the minimum inhibitory concentration (MIC) of cMoL was determined using the microdilution assay proposed by G. R. S. Ferreira et al. (2023). In 96‐well microtiter plates, 80‐μL cMoL was added to the third well, from which it was serially diluted using sterile distilled water (80 μL) to reach concentrations of 30.0, 15.0, 7.5, 3.75, 1.85, and 0.94 μg/mL. Fluconazole (0.125–64 μg/mL) was used as the positive control. Subsequently, 40 μL of Sabouraud dextrose broth (SDB) was added to all wells, except to the first one, which was filled with 200‐μL culture medium, corresponding to the sterility control. Next, all wells (except the sterility control) were inoculated with 80‐μL fungal culture in SDB (3 × 10^6^ CFU/mL). The second well corresponded to the 100% growth control. The optical density was measured at 600 nm (OD_600_) before and after incubation for 48 h at 30°C. The minimal inhibitory concentration (MIC_50_) was determined as the lowest cMoL concentration able to inhibit fungal growth by at least 50% in comparison with the 100% growth control. The minimal fungicidal concentration (MFC) was checked by inoculating SDA plates with the well contents (10 μL) containing cells exposed to cMoL at concentrations higher than or equal to the MIC. The plates were incubated at 30°C for 48 h, and the MFC corresponded to the lowest concentration of cMoL able to reduce the number of CFU by 99.9% compared with the initial inoculum. Three independent experiments were performed in triplicates.

#### 2.2.3 Cell Death Evaluation by Flow Cytometry

For cell death evaluation, yeast was cultured for 48 h at 30°C in the absence (negative control) or presence of cMoL (MIC_50_) under the same conditions as previously described. The FITC Annexin V Apoptosis Detection Kit I (BD Biosciences, San Jose, CA, USA) was used to assess cell death according to the manufacturer's instructions. Addition of isopropyl alcohol (20%, v/v) to the untreated fungal cells was used as a positive control. A gate was created to exclude cell debris and define the cell population of interest. Data were acquired using a BD Accuri C6 cytometer (BD Biosciences), and a minimum of 20,000 events at the gate were collected per sample. An FL1 filter (excitation 488 nm, emission 530/30 nm) was used for Annexin V (AnnV)‐FITC detection, and an FL3 filter (excitation 488 nm, emission 585/40 nm) was used for propidium iodide (PI) detection. The FL1 and FL3 quadrants were established based on the intrinsic fluorescence of cells from the negative control unstained with AnnV‐FITC or PI. Negative control cells stained with AnnV‐FITC or PI alone were used to set the compensation parameters. Cells stained only with annexin (AnnV+/PI−) were considered apoptotic, those stained with PI alone (AnnV−/PI+) were considered necrotic, those stained with both probes (AnnV+/PI+) were in late apoptosis or necrosis, and unstained cells (AnnV−/PI−) were considered viable. Each assay was performed in triplicate, and two independent experiments were performed. The results were obtained using the BD Accuri C6 software (BD Biosciences).

#### 2.2.4 Antibiofilm Assay

The antibiofilm activity of cMoL was assessed according to the protocol described by P. M. Silva et al. (2018). *C. neoformans* (B3501 and H99) and *C. gattii* (R265) were cultured in SDB overnight at 37°C and subsequently suspended in sterile 0.9% (w/v) NaCl solution to obtain a fungal suspension with a concentration of 10^8^ CFU/mL. Each well of a 96‐well microplate received 40‐μL SDB and 80‐μL fungal suspension. Subsequently, 80 μL of ultrapure Milli‐Q water (control) or cMoL (12.5–100 μg/mL) was added. OD_600_ was measured at time zero and after 48 h of incubation at 30°C. Following incubation, the culture medium was discarded, and the wells were washed three times with 0.15‐M NaCl. The fungal cells were heat fixed at 60°C for 40 min, and biofilm formation was assessed by staining with 0.4% (w/v) crystal violet for 15 min at 25°C. The wells were then washed with water, and the stain was solubilized with ethanol for 15 min. The absorbance was recorded at 570 nm. Three independent experiments were conducted in triplicates.

#### 2.2.5 Synergism Assessment

The synergistic effect of cMoL and the commercial antibiotic fluconazole was evaluated according to the procedure described by Pillai et al. (2005). SDB (100 μL) was added to each well of a 96‐well microplate. cMoL (100 μL) was added to the well at a serially diluted concentration (0.97–800 μg/mL). Thereafter, the antibiotic solution (100 μL; 4 × MIC) was serially added in the reverse direction to which cMoL was added. A negative control was performed in another row and corresponded to 100% of fungal growth. After dilution, suspensions of *C. neoformans* (B3501 or H99) and *C. gattii* (R265) were added to the wells, and the assay was continued as described above to determine the MIC_50_. To assess the interaction between the different treatments, the fractional inhibitory concentration index (FICi) values were calculated from the sum of the MIC ratios of cMoL or fluconazole tested separately and in combination. The following classification was then used: FICi ≤ 0.5 indicates synergism; 0.5 < FICi ≤ 1 indicates an additive effect; 1 < FICi ≤ 2 indicates no effect; and FICi > 2 indicates an antagonistic effect.

### 2.3 Toxicity Assessment

#### 2.3.1 Animals

Sixty‐day‐old female Swiss albino mice weighing 25–30 g were obtained from the vivarium of the *Instituto Keizo Asami* (iLIKA) of the UFPE. Animals were fed with a standard diet ad libitum and kept under standardized temperature (22°C ± 2°C), light/dark cycle (12 h/12 h), and humidity (50%–60%). The experimental protocols were approved by the Ethics Committee on the Use of Animals at UFPE (Protocol No. 140/2022).

#### 2.3.2 Acute Toxicity Assay

cMoL was evaluated for acute toxicity after oral or intraperitoneal administration. A dose of 200 mg/kg was selected based on previous studies of in vivo toxicity of plant proteins, which evaluated doses between 100 and 300 mg/kg (A. R. Silva et al. 2022; Patriota et al. 2021). Mice previously fasted for 4 h were divided into four groups (three animals/group): Group 1 (control—intraperitoneal) was treated with 0.15‐M NaCl ip, Group 2 was treated with 200‐mg/kg cMoL ip, Group 3 (control—gavage) was treated per os with 0.15‐M NaCl, and Group 4 was treated with 200‐mg/kg cMoL per os. Sterile (autoclaved) saline solution (0.15‐M NaCl) was used in the control groups and for cMoL dilution, and the treatment volume for each animal was 0.1 mL per 10 g of body weight (0.1 mL/10 g). Two independent experiments were performed. In the first 2 h after administration, the animals were evaluated for behavioral signs of toxicity, such as diarrhea, piloerection, tachycardia, or death. LD_50_ was defined as the dose that promoted 50% of animals' death. The animals were evaluated daily for 14 days for body weight and water and food consumption. At the end of Day 14, the mice were anesthetized with 77.3‐mg/kg ketamine and 13.3‐mg/kg xylazine ip for blood collection (1 mL) via hepatic vein puncture. After blood collection, the animals were euthanized using 300‐mg/kg ketamine and 30‐mg/kg xylazine, and the spleen, heart, liver, lungs, and kidneys were collected for macroscopic and histological analyses. If an animal showed signs of toxicity corresponding to humane endpoints (pronounced weight loss, dehydration, loss of walking ability, inability to access food or water, and labored respiration) before the end of the test, it was euthanized immediately.

##### 2.3.2.1 Biochemical and Hematological Parameters

For the analysis of hematological parameters, part of the collected blood was transferred to a 0.5‐mL microtube containing EDTA K3 (FirstLab, Paraná, Brazil) and evaluated using a Sysmex XP‐300 automatic hematology analyzer to determine erythrocytes, hemoglobin, hematocrit, mean corpuscular volume (MCV), mean corpuscular hemoglobin (MCH), mean corpuscular hemoglobin concentration (MCHC), total leukocytes, and platelets. Differential leukocyte counts were performed using blood smears stained with rapid panoptic under a Nikon E100 optical microscope. Another portion of the blood was transferred to a 0.5‐mL microtube containing a clot activator and separator gel (FirstLab, Paraná, Brazil) and centrifuged (1000 *g*; 10 min) to obtain the serum. The serum biochemical parameters were evaluated using specific kits (Labtest Diagnóstica, Lagoa Santa, Brazil). Total proteins (Labtest Diagnóstica Kit 99), urea (Labtest Diagnóstica Kit 27), creatinine (Labtest Diagnóstica Kit 96), glucose (Labtest Diagnóstica Kit 133), total cholesterol (Labtest Diagnóstica Kit 76), and triglycerides (Labtest Diagnóstica Kit 87) levels (mg/dL) were determined colorimetrically in relation to their respective standards according to the manufacturer's instructions. The activities (U/L) of alanine aminotransferase (ALT) (Labtest Diagnóstica Kit 108), aspartate aminotransferase (AST) (Labtest Diagnóstica Kit 109), and gamma‐glutamyl transferase (GGT) (Labtest Diagnóstica Kit 105) were determined using the kinetic‐ultraviolet method according to the manufacturer's instructions.

##### 2.3.2.2 Cytokine Level Determination

Cytokine levels were measured to determine whether cMoL treatment elicited an inflammatory response after 14 days. An aliquot of the serum samples, obtained as described previously, was subjected to cytokine quantification using the BD Cytometric Bead Array (CBA) Mouse Th1/Th2/Th17 kit (Becton Dickinson Biosciences, USA) according to the manufacturer's instructions. The levels of the interleukins IL‐2, IL‐4, IL‐6, IL‐10, and IL‐17A; tumor necrosis factor (TNF‐α); and interferon‐gamma (IFN‐γ) were evaluated. The data were collected using FL2 and FL4 filters, with thresholds of 500,000 in both FSC and SSC, acquired on a BD Accuri C6 cytometer, and the results were analyzed using the BD Accuri C6 software.

##### 2.3.2.3 Histological Analyses

Section of the collected organs (spleen, heart, liver, lungs, and kidneys) was fixed in buffered formalin (10% v/v), dehydrated in ethanol, diaphanized in xylol, and embedded in paraffin. Histological sections (5 μm) were obtained using a microtome, stained with hematoxylin–eosin, and mounted with synthetic resin and coverslips. Histopathological analysis was performed using the ZEN blue edition image capture system and Axio Zeiss microscope equipped with a microscope camera (Zeiss AXIOCAMERc5s). To compare structural changes, abnormalities in the tissue sections were graded from 0 (normal structure) to 3 (severe pathological changes) (Ibrahim et al. 2018) by two independent pathologists. A slide was prepared for each organ collected from each animal from each experimental group.

#### 2.3.3 Genotoxicity Assessment

##### 2.3.3.1 Comet Assay

The mice (*n* = 5 animals/group) were divided into the same four groups as the acute toxicity assay, with the addition of a positive control group that received 20‐mg/kg methotrexate (MTX) via intraperitoneal injection. Twenty‐four hours after the treatments, an aliquot of whole blood (60 μL) was collected from the tail via venipuncture, and the alkaline version of the comet assay was performed according to the procedures described by Barros et al. (2022). A mixture composed of whole blood (20 μL) and 0.5% low‐melting‐point agarose (110 μL, 37°C) was deposited on microscope slides previously covered with standard agarose (1.5%) and covered with a cover slip. Thereafter, the slides were immersed in a lysis solution (2.5‐M NaCl, 100‐mM EDTA, and 10‐mM Tris) for 1 h at 4°C. Slides were transferred into an electrophoresis vessel and covered with running buffer (300‐mM NaOH and 1‐mM EDTA, pH 13) for 20 min. Electrophoresis was performed for 20 min at 32 V and a current of 300 mA. Subsequently, the slides were covered with a neutralization solution (0.4‐M Tris, pH 7.5) for 15 min and fixed with absolute ethanol for 5 min. Each slide was stained with PI and analyzed under a fluorescence microscope (Zeiss Imager M2; Carl Zeiss AG, Jena, Germany). One hundred nucleoids were evaluated per slide, and the relationship between tail length and comet head size was observed. Nucleoids were classified into the following five classes: Class 0, no tail and no damage; Class 1, tail length smaller than the head diameter; Class 2, tail length between one and two times the head diameter; Class 3, tail length greater than twice the head diameter; and Class 4, absence of the head (Costa and Teixeira 2014). Three independent experiments were performed.

The damage index (*DI*) was calculated using the following formula (*n* = number of cells in each class) (Costa and Teixeira 2014):

$$\mathrm{DI}=\left(0\times n_{0}\right)+\left(1\times n_{1}\right)+\left(2\times n_{2}\right)+\left(3\times n_{3}\right)+\left(4\times n_{4}\right).$$

The damage frequency (*DF*, %) was calculated based on the number of cells without damage (Class 0) in relation to the total nucleoids observed in each treatment, using the following formula (Costa and Teixeira 2014):

$$\mathrm{DF}=\frac{\left(\left[\text{total nucleoids}-\text{number of Class}\;0\;\text{nucleoids}\right]\times 100\right)}{\text{total nucleoids}}.$$

##### 2.3.3.2 Micronucleus Test

For the micronucleus test, blood samples were collected from the same animals as in the comet assay, but 48 h after the treatment. Before the test, microscope slides were washed with distilled water, bathed in 70% alcohol, and placed at 80°C. Warm slides were stained with acridine orange (1 mg/mL) using a glass rod and dried at room temperature (25°C). Subsequently, 5 μL of total blood from each animal was deposited on the slides and then covered with a coverslip (Eiji et al. 1992). The slides were analyzed at 40× magnification using a Zeiss Imager M2 fluorescence microscope (Alexa Fluor 488 filter). The presence of micronuclei was analyzed in 2000 polychromatic erythrocytes per animal. The results are expressed as the number of micronucleated polychromatic erythrocytes (MnPCE).

### 2.4 Statistical Analysis

The results from flow cytometry and toxicity analysis are represented by mean ± standard error of the mean (SEM). Data normality was evaluated using the Shapiro–Wilk test. Data were analyzed by one‐way analysis of variance (ANOVA) and Tukey's post‐test using Prism GraphPad 7.0 Software (GraphPad Software, San Diego, CA, USA) and *p* values ≤ 0.05 were considered statistically significant.

## 3 Results and Discussion

### 3.1 Antifungal Activity

**FIGURE 1:**
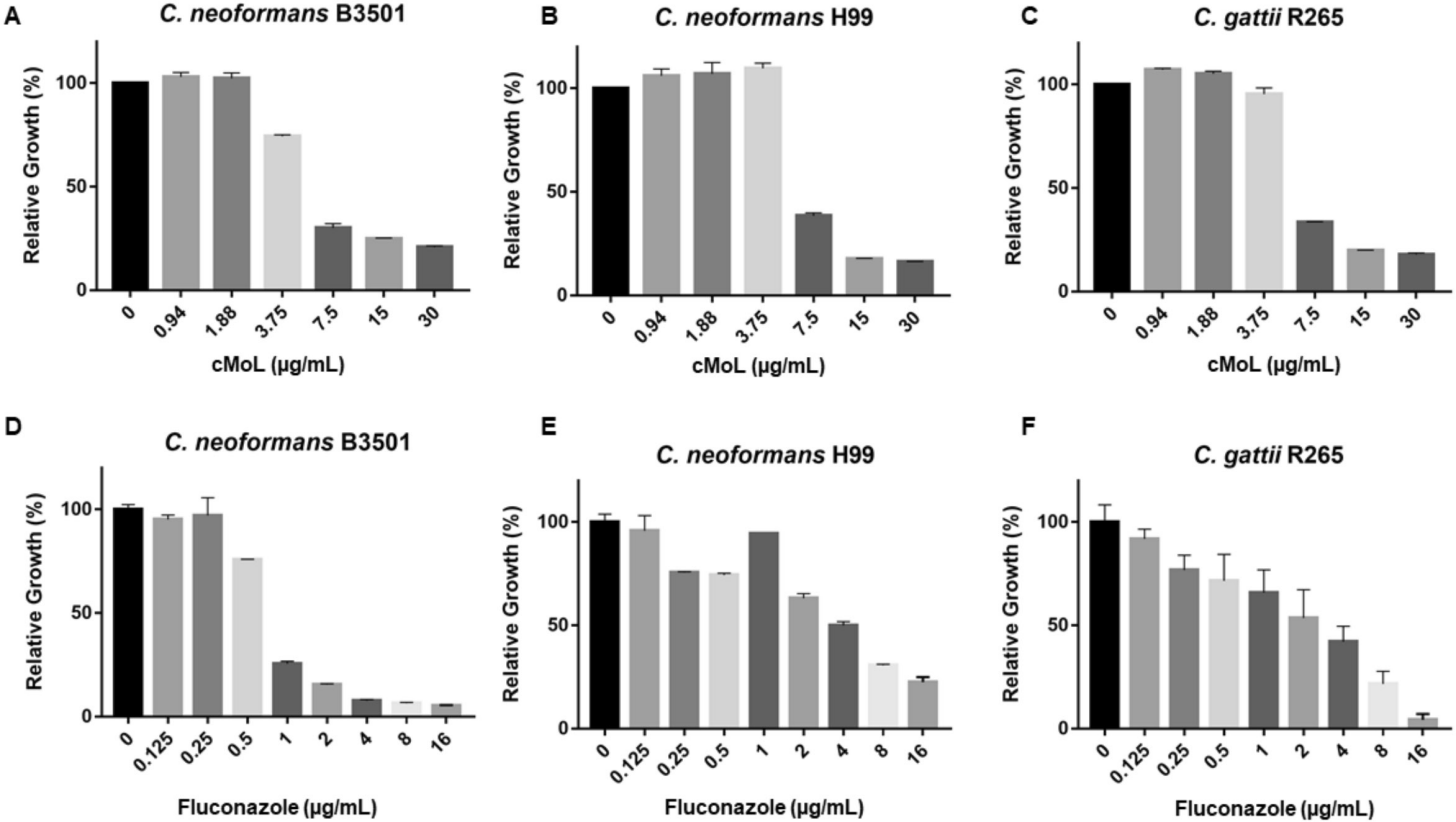
Percentage of relative growth of *Cryptococcus neoformans* B5301 (A,D) or H99 (B,E) and *Cryptococcus gattii* R265 (C,F) treated with different concentrations (0.94–30 μg/mL) of the coagulant *Moringa oleifera* lectin (cMoL) or fluconazole (0.125–16 μg/mL) for 48 h, as compared to negative control (100% growth).

**FIGURE 2:**
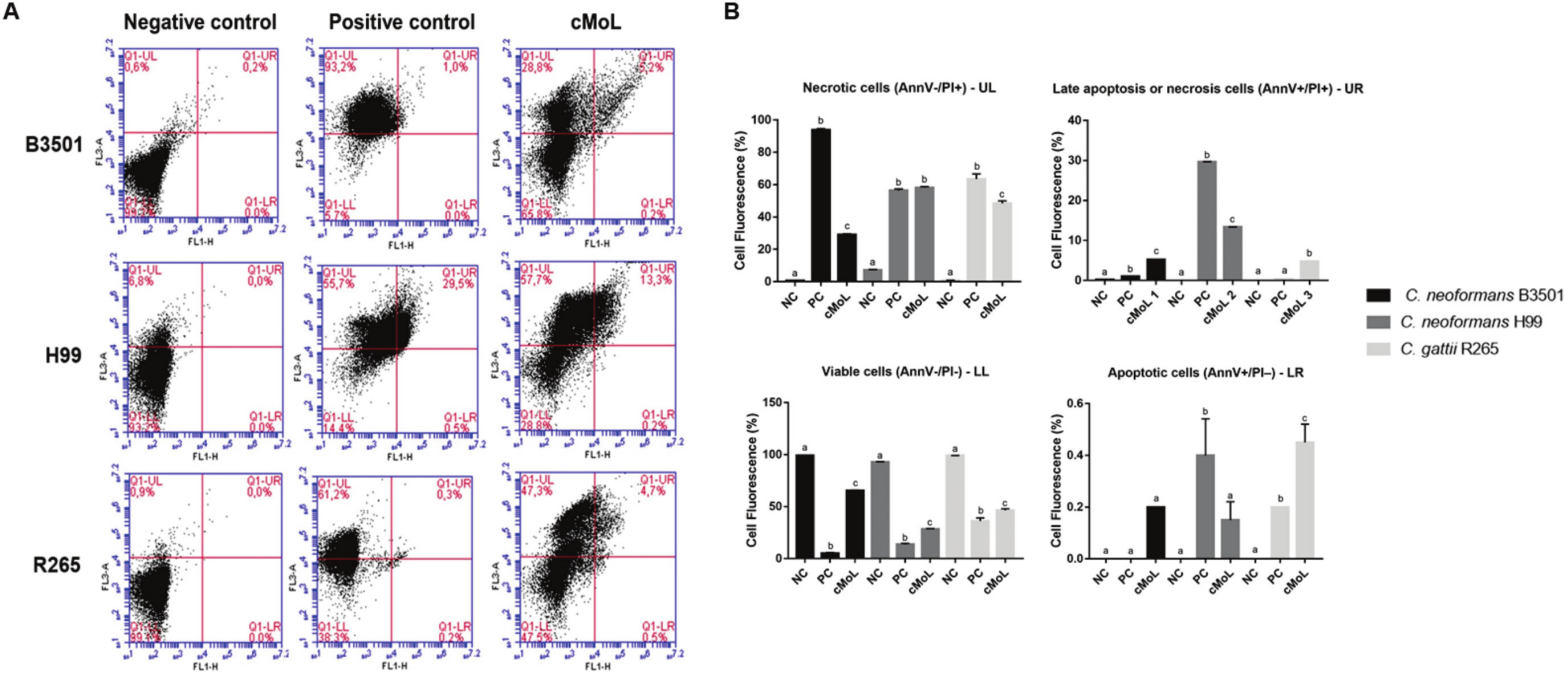
Investigation of cell death in *Cryptococcus neoformans* B5301 or H99 and *Cryptococcus gattii* R265 untreated (negative control) or treated with cMoL for 48 h at 7.5 μg/mL or 20% isopropyl alcohol (positive control). (A) Cell death assessed by flow cytometry using Annexin V (AnnV) and propidium iodide (PI). The images are representative FL1 filter (stained with AnnV) versus FL3 filter (stained with PI) dot plots. (B) Cell fluorescent percentual (%) where events corresponding to apoptotic (Ann+) or necrotic (PI+) cells can be seen in the LR and UL quadrants, respectively. Cells in late apoptosis or necrosis (AnnV+/PI+) are represented in the UR quadrant. Viable cells are in the LL quadrant. The data corresponded to the mean ± SD.

cMoL showed MIC_50_ of 7.5 μg/mL for *C. neoformans* (B3501 and H99) and *C. gattii* because more than 50% inhibition of the fungal growth was observed at this concentration (Figure 1A–C). Fungicidal effects were not observed. The fluconazole (positive control) presented a MIC_50_ of 1 μg/mL for *C. neoformans* B3501 and 8 μg/mL for *C. neoformans* H99 and *C. gattii* R265 (Figure 1D–F). Investigation of *Cryptococcus* cell death by AnnV and PI staining and flow cytometry (Figure 2) revealed that cMoL at MIC_50_ concentration promoted necrosis and apoptosis in all tested species. The strongest effect was observed for *C. neoformans* H99 (28.35% ± 0.45% of viable cells) and *C. gattii* R265 (47.3% ± 1.1% of viable cells). cMoL‐treated *C. neoformans* B3501 cells showed 65.45% ± 0.35% of viability.

Lectins display antifungal activity by interacting with carbohydrates on fungal cell wall components, leading to growth inhibition, reduced nutrient absorption, altered spore germination, and cell death (P. M. Silva et al. 2018; A. R. Silva et al. 2021). For example, the recombinant mannose‐binding lectin Scytovirin shows fungicidal potential against *C. neoformans* B3501 through its interaction with the cell wall, affecting the size and release of the fungal capsule (Jones et al. 2017). PgTeL (a chitin‐binding lectin isolated from the sarcotesta of *Punica granatum* ) showed fungistatic activity on *C. neoformans* B3501 Serotype D (MIC_50_ = 172.0 μg/mL) with MIC_50_ 23 times higher in relation to that determined for cMoL (G. R. S. Ferreira et al. 2023). In addition, cMoL was more efficient than ConBr II (a d‐mannose‐binding lectin from *Canavalia brasiliensis* seed), which presented MIC > 500 μg/mL against *Cryptococcus* spp. (Klafke et al. 2013).

The interaction of lectins with the components present in the cell wall of *Cryptococcu*s was also demonstrated in a study conducted using conjugates of PgTeL with quantum dots, which intensely marked *C. neoformans* cell surface (A. R. Silva et al. 2022). The capsule of *Cryptococcus* is composed of glucuronoxylomannan and glucuronoxylomannogalactan, whereas the cell wall is a carbohydrate‐rich structure formed by α‐1,3‐glucan, β‐1,3‐glucan, β‐1,6‐glucan, chitin, chitosan, and proteins (Z. A. Wang, Li, and Doering 2018). *N*‐acetyl‐d‐glucosamine is highly expressed on the *C. neoformans* cell wall surface, and methyl‐α‐d‐mannoside, d‐galactose, and l‐fucose are also detected (Ximenes et al. 2015). Previous studies have shown that cMoL binds to galactose, d‐glucose, and glycoproteins; therefore, the antifungal activity exhibited by cMoL is probably related to its interaction with cell wall components (A. F. Santos et al. 2009).

**FIGURE 3:**
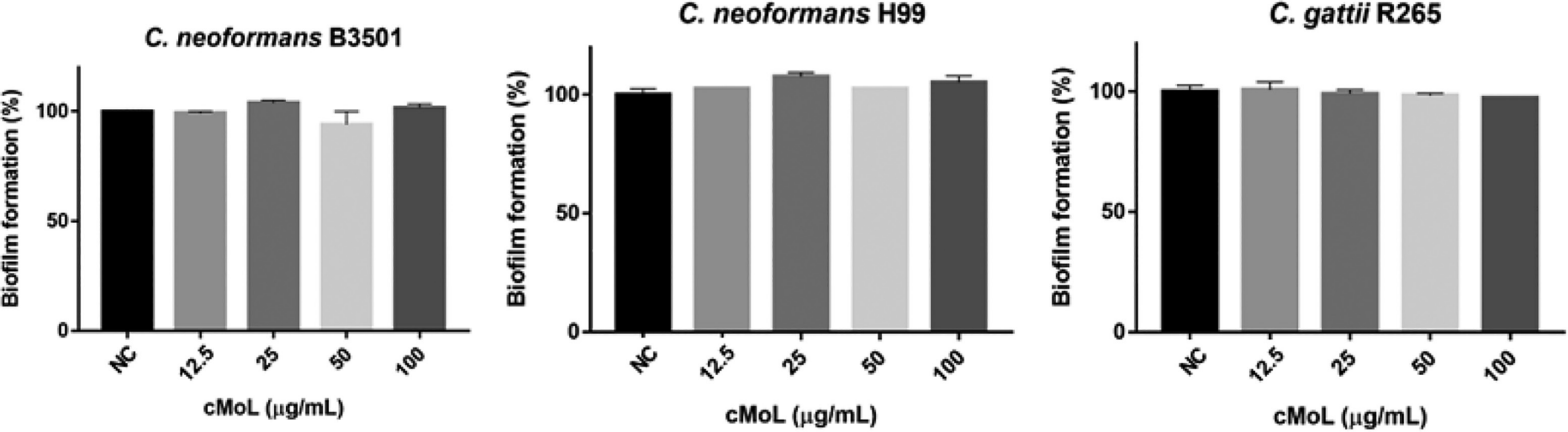
Percentage biofilm formation by *Cryptococcus neoformans* B3501 or H99 and *Cryptococcus gattii* R265 treated for 48 h with different concentrations (12.5–100 μg/mL) of cMoL. NC: negative control.

During the antibiofilm assay, cMoL could not reduce biofilm formation by *Cryptococcus* isolates (Figure 3). Biofilms are structured assemblages of microorganisms inserted into a self‐synthesized extracellular matrix composed of exopolysaccharides, proteins, teichoic acids, enzymes, and extracellular DNA (Klein et al. 2015). Biofilm formation is a significant virulence factor that is directly associated with the increased resistance of fungal cells, as it hinders the penetration of antifungal agents and limits their action (Moura et al. 2017). The *Cratylia floribunda* lectin (CFL) in concentrations between 31.25 and 250 μg/mL moderately inhibits the growth of *Candida albicans* planktonic cells; however, these same concentrations are not able to reduce the yeast biofilm formation (Vasconcelos et al. 2014). In contrast to the absence of antibiofilm activity of cMoL, water‐soluble *M. oleifera* lectin (WSMoL), another lectin purified from *M. oleifera* seeds, is able to reduce biofilm formation by *C. neoformans* B3501 (L. M. M. Santos et al. 2024).

Considering the fungistatic activity of cMoL, a synergy assay with the commercial drug fluconazole was conducted to assess whether cMoL could enhance antibiotic activity (Table 1). cMoL decreased the MIC_50_ of fluconazole against *C. neoformans* H99 cells, indicating a synergistic effect. An antagonistic effect was observed in *C. neoformans* B3501 and *C. gattii* R265. The observed synergistic effect is important from a health point of view, as H99 is among the *C. neoformans* varieties with the highest worldwide distribution and causes death due to meningoencephalitis (Chen et al. 2014; Martinez et al. 2001). Lectins can enhance the action of antibiotics by interacting with carbohydrates on the cell membrane and facilitating drug entry, or by binding to glycans present in efflux pumps, thereby inhibiting or altering their structural conformation (V. F. Santos, Araujo, et al. 2020; Duarte et al. 2023). Even with their fungistatic or fungicidal activities, lectins may or may not have synergistic effects. PgTeL showed no effect when used in combination with amphotericin B against *C. neoformans* B3501 (G. R. S. Ferreira et al. 2023). The *Myracrodruon urundeuva* heartwood lectin (MuHL) at 25 μg/mL decreased the MIC_50_ of fluconazole by 16 times against *C. neoformans* B3501 (Videres et al. 2023).

**TABLE 1:** Synergy assessment of coagulant *Moringa oleifera* lectin (cMoL) in combination with fluconazole against *Cryptococcus* isolates.

Fungi	MIC_50_ (μg/mL)		MIC_50_ (μg/mL)—Combination		FICi	Effect
	cMoL	Fluconazole	cMoL	Fluconazole		
*Cryptococcus neoformans* (H99)	7.5	8	0.097	2	0.26	Synergism
*Cryptococcus neoformans* (B3501)	7.5	1	0.097	2	2.01	Antagonism
*Cryptococcus gattii* (R265)	7.5	8	800	16	108.67	Antagonism

Abbreviations: FICi: fractional inhibitory concentration index; ND: not detected.

### 3.2 Acute Toxicity

Toxicity assessment is a key factor in the biomedical employment of lectins, because the promotion of undesired effects can limit their application. The treatment with cMoL (200 mg/kg per os and ip) did not cause death; therefore, the lethal dose that causes 50% of death (LD_50_) is greater than 200 mg/kg (LD_50_ > 200 mg/kg). The treatments did not cause changes in the weight of the animals or in the consumption of water and food during the 14 days of observation compared to the negative controls (Table 2). The absence of cMoL toxicity in mice is in contrast with the effects of other lectins reported in the literature. WSMoL caused 60% of mice mortality in an acute toxicity test at 200 mg/kg ip (J. S. Brito et al. 2023). After 6 days of dosing in mice, *Agrocybe aegerita* lectin (25, 50, and 250 mg/kg per os) significantly decreased body weight, increased ALT/AST levels, promoted partial liver necrosis, and caused content loss mainly in the hepatocytes (Jin et al. 2014). The lectin from *Viscum album* administered intravenously in mice presented LD_50_ between 50 and 100 μg/kg (Lin et al. 2010), whereas the lectin from *Jatropha curcas* seeds (curcin) was acutely toxic to mice with oral LD_50_ of 104.74 ± 29.45 mg/kg (Barbieri et al. 1993). Additionally, ricin ( *Ricinus communis* lectin) is a known toxic protein whose effects depend on the dose and administration route, with an LD_50_ of 2.6 mg/kg via parenteral administration and 29.0 mg/kg via oral administration (X. He et al. 2010; A. J. C. A. Santos, Barros, et al. 2020).

**TABLE 2:** Water (mL/animal/day) and food (g/animal/day) consumption and body weight gain (g) of female Swiss albino mice that received orally or intraperitoneally saline solution (negative control) or the coagulant *Moringa oleifera* lectin (cMoL) at 200 mg/kg.

Parameters	NC (oral)	cMoL (oral)	*p*	NC (ip)	cMoL (ip)	*p*
Weight gain (g)	1.67 ± 0.89	0.0 ± 0.58	0.3228	0.33 ± 0.67	1.67 ± 0.88	0.5041
Water consumption (mL/animal/day)	7.5 ± 0.34	6.67 ± 0.14	0.0622	6.64 ± 0.28	6.67 ± 0.2	> 0.9999
Food consumption (g/animal/day)	6.68 ± 0.18	6.49 ± 0.15	> 0.9999	6.69 ± 0.21	6.7 ± 0.27	> 0.9999

*Note:* Values represented by mean ± SEM (*n* = 3/group). Weight gain was determined by the difference between initial and final weight for each animal. No significant differences (*p* > 0.05) were found between cMoL‐treated group and the respective control (oral or ip).

Abbreviation: NC: negative control.

Regarding hematological parameters, the levels of erythrocytes, hemoglobin, hematocrit, MCV, MCH, MCHC, leukocytes, and platelets did not differ (*p* > 0.05) from those observed in the saline‐treated controls (Table 3). Biochemical analysis of blood did not indicate significant changes (*p* > 0.05) in the levels of total proteins, urea, creatinine, glucose, total cholesterol, triglycerides, ALT, AST, or GGT in the negative controls (Table 4). Some plant lectins have been shown to alter hematological and biochemical parameters in toxicology studies in vivo. For example, the phytotoxin Abrin (LD_50_: 0.91 μg/kg ip) obtained from the seeds of the *Abrus precatorius* plant altered hemoglobin and white and red blood cell counts significantly (Chaturvedi et al. 2015). PgTeL increased HDL levels and decreased triglyceride, LDL, and LDL levels in an in vivo acute toxicity assay (Prasanna and Venkatesh 2015).

**TABLE 3:** Hematological parameters of female Swiss albino mice that received orally or intraperitoneally saline solution (negative control) or the coagulant *Moringa oleifera* lectin (cMoL) at 200 mg/kg.

Parameter	NC (oral)	cMoL (oral)	*p*	NC (ip)	cMoL (ip)	*p*
Erythrocytes (10^6^/mm^3^)	7.33 ± 0.34	7.63 ± 0.38	> 0.9999	7.55 ± 0.35	7.66 ± 0.33	> 0.9999
Hematocrit (%)	38.3 ± 1.35	40.4 ± 1.88	0.7701	39.1 ± 1.5	40.1 ± 1.78	> 0.9999
Hemoglobin (g/dL)	11.9 ± 0.30	12.8 ± 0.53	0.4538	12.9 ± 0.48	13.0 ± 0.58	> 0.9999
MCV (fL)	52.2 ± 0.64	53.0 ± 0.25	0.5854	51.8 ± 0.42	52.3 ± 0.53	0.9672
MCH (pg)	16.3 ± 0.35	16.8 ± 0.17	0.3481	17.1 ± 0.2	16.9 ± 0.25	> 0.9999
MCHC (%)	31.1 ± 0.43	31.7 ± 0.2	0.3928	33.0 ± 0.26	32.3 ± 0.19	0.2397
Leukocytes (10^3^/mm^3^)	6.03 ± 1.28	12.2 ± 1.58	0.1347	7.6 ± 1.55	9.63 ± 3.27	> 0.9999
Segmented (%)	17.3 ± 6.96	8.67 ± 1.2	0.4785	12.7 ± 3.38	14.3 ± 2.73	> 0.9999
Lymphocytes (%)	57.0 ± 7.0	68.3 ± 5.24	0.3554	65.3 ± 8.84	62.0 ± 2.08	> 0.9999
Monocytes (%)	17.3 ± 1.45	9.33 ± 1.67	0.0592	11.7 ± 2.03	16.0 ± 3.06	0.3805
Eosinophils (%)	1.67 ± 1.20	0.67 ± 0.33	0.6932	0 ± 0	1.67 ± 0.67	0.2683
Basophils (%)	3.0 ± 1.73	11.7 ± 4.18	0.1750	10.3 ± 3.67	6.33 ± 2.4	0.7906
Platelets (10^6^/mm^3^)	1.67 ± 1.2	0.67 ± 0.33	0.5850	0 ± 0	1.67 ± 0.67	0.2776

*Note:* Values represented by mean ± SEM (*n* = 3/group). No significant differences (*p* > 0.05) were found between cMoL‐treated group and the respective control (oral or ip).

Abbreviations: MCH: mean corpuscular hemoglobin; MCHC: mean corpuscular hemoglobin concentration; MCV: mean corpuscular volume; NC: negative control.

**TABLE 4:** Blood biochemical parameters of female Swiss albino mice that received orally or intraperitoneally saline solution (negative control) or the coagulant *Moringa oleifera* lectin (cMoL) at 200 mg/kg.

Parameter	NC (oral)	cMoL (oral)	*p*	NC (ip)	cMoL (ip)	*p*
Total protein (mg/dL)	4.22 ± 0.12	4.02 ± 0.17	0.4931	4.29 ± 0.09	4.5 ± 0.04	0.4654
Glucose (mg/dL)	176.0 ± 22.09	132.88 ± 14.46	0.1953	145.11 ± 13.12	194.8 ± 13.74	0.1254
Triglycerides (mg/dL)	153.06 ± 7.24	152.38 ± 7.36	> 0.9999	176.87 ± 13.62	151.02 ± 16.59	0.3267
Total cholesterol (mg/dL)	115.3 ± 4.19	102.09 ± 7.59	0.8365	135.27 ± 19.19	106.6 ± 5.96	0.2023
ALT (U/L)	42.56 ± 1.56	29.65 ± 8.22	0.3742	37.22 ± 5.84	47.55 ± 7.49	0.5634
AST (U/L)	52.19 ± 0.58	58.53 ± 7.65	> 0.9999	63.43 ± 7.28	51.32 ± 7.74	0.4550
GGT (U/L)	9.45 ± 2.39	7.73 ± 0.74	> 0.9999	5.58 ± 0.43	9.88 ± 7.3	0.9094
Creatinine (mg/dL)	0.33 ± 0.11	0.33 ± 0.22	> 0.9999	0.38 ± 0.19	0.71 ± 0.17	0.4383
Urea (mg/dL)	59.1 ± 0.10	62.11 ± 2.99	0.6324	64.53 ± 0.50	59.02 ± 2.59	0.1733

*Note:* Values represented by mean ± SEM (*n* = 3/group). No significant differences (p > 0.05) were found between cMoL‐treated group and the respective control (oral or ip).

Abbreviations: ALT: alanine aminotransferase; AST: aspartate aminotransferase; GGT: gamma‐glutamyl transferase; NC: negative control.

Regarding cytokine release, oral treatment with cMoL did not cause significant changes compared to the negative control (Table 5). Lectins can exert immunomodulatory effects by binding to glycans present on the surface of immune cells and stimulating or suppressing cytokine production (A. J. C. A. Santos, Barros, et al. 2020). The onion lectin *Allium cepa* agglutinin (ACA) significantly stimulated the production of pro‐inflammatory cytokines (TNF‐α and IL‐12) and macrophages (P. K. M. O. Brito et al. 2017). The presence of *Schinus terebinthifolia* leaf lectin (SteLL) stimulated mice splenocytes to release pro‐inflammatory cytokines (IL‐17A, TNF‐α, IFN‐γ, and IL‐2) and IL‐4, an anti‐inflammatory cytokine that can prevent exacerbated inflammation (A. J. C. A. Santos, Barros, et al. 2020). However, some plant lectins, such as cMoL, do not influence the immune system of animals. For example, the subcutaneous administration of ArtinM ( *Artocarpus heterophyllus* lectin) in infected mice did not affect the production of IL‐12, IFN‐γ, IL‐10, or TNF‐α by the liver, spleen, lungs, kidneys, and heart (Yamamoto et al. 2013).

**TABLE 5:** Cytokine levels (pg/mL) in the serum of female Swiss albino mice that received orally or intraperitoneally saline solution (negative control) or the coagulant *Moringa oleifera* lectin (cMoL) at 200 mg/kg.

Parameters	NC (oral)	cMoL (oral)	*p*	NC (ip)	cMoL (ip)	*p*
IL‐2	51.5 ± 6.02	35.9 ± 0.8	0.0169	47.7 ± 4.41	39.2 ± 3.86	0.2581
IL‐4	41.5 ± 2.79	29.2 ± 4.47	0.1684	43.4 ± 2.87	53.2 ± 6.49	0.3116
IL‐6	30.1 ± 1.03	24.9 ± 0.55	0.4022	26.7 ± 4.75	26.6 ± 1.85	> 0.9999
IL‐10	26.3 ± 0.98	18.6 ± 1.1	0.0751	32.8 ± 3.62	33.1 ± 1.99	> 0.9999
IL‐17	14.8 ± 0.73	18.1 ± 1.68	0.3140	20.2 ± 1.47	24.3 ± 1.84	0.1799
INF‐γ	75.10 ± 2.96	63.08 ± 2.40	0.0732	62.64 ± 5.28	71.32 ± 1.89	0.2154
TNF‐α	22.1 ± 0.63	23.4 ± 0.37	> 0.9999	18.1 ± 2.91	19.8 ± 1.8	> 0.9999

*Note:* Values represented by mean ± SEM (*n* = 3/group). No significant differences (*p* > 0.05) were found between cMoL‐treated group and the respective control (oral or ip).

Abbreviations: IFN: interferon; IL: interleukin; NC: negative control; TNF: tumor necrosis factor.

**FIGURE 4:**
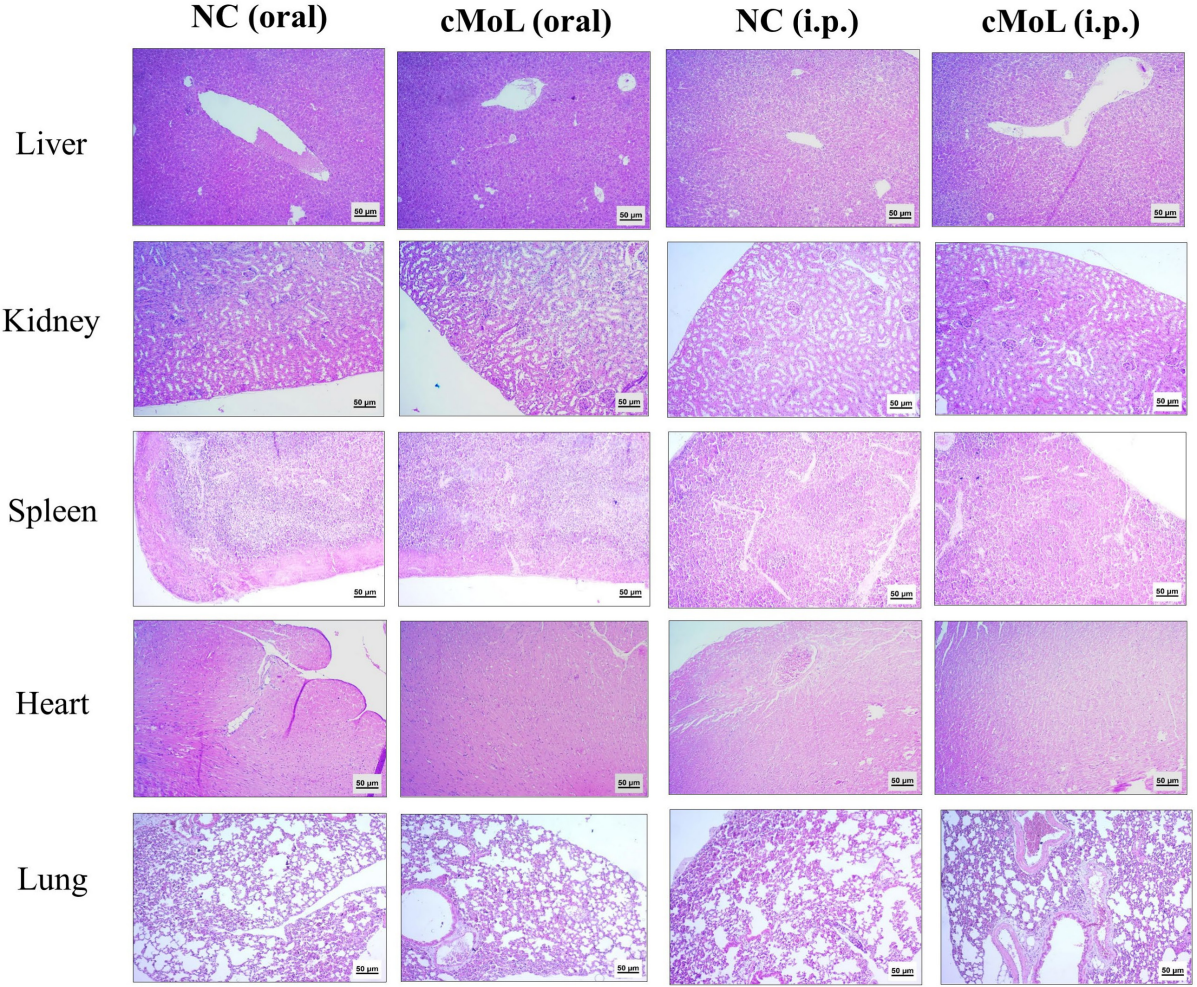
Histopathological analysis of the liver, spleen, kidney, heart, and lung from animals that received saline solution (negative control) or a single dose (200 mg/kg) of cMoL orally or intraperitoneally 14 days before collection of the organs. In all groups, no signs of tissue alteration were observed.

The toxic mechanisms of lectins may be related to dramatic changes in cellular morphology and metabolism caused by their carbohydrate‐recognition properties, which can be observed through macroscopic and histopathological studies (Dang and Damme 2015; Mehrvar et al. 2021). No differences in relation to the negative controls were observed in the weights of the liver, kidneys, spleen, heart, and lungs of animals subjected to oral or intraperitoneal cMoL treatments (Table 6). Histopathological data from toxicological studies are critical during the drug development process, as they allow for a detailed analysis of the cellular and structural changes that occur in response to exposure to toxic substances (Poduri et al. 2013). Histopathological analysis of the organs did not indicate signs of toxicity in either administration route with cMoL compared to the negative control. The livers of the animals (Figure 4) were well preserved, with no steatosis processes, inflammatory infiltrates, or molecular deposits (damage score = 0). The kidneys (Figure 3E–H) presented nephrons that maintained their structural homogeneity, well‐preserved tubules, and an absence of inflammatory infiltrates or molecular deposits (damage score = 0). The spleens (Figure 4) were preserved, including the capsule, white pulp (evidence of lymphoid follicles), preserved red pulp, and the absence of congestive processes, reactivity, or macrophage changes (damage score = 0). The heart (Figure 4) did not have changes in cardiac muscle fibers, absence of inflammatory changes, or presence of molecular deposits (damage score = 0). Finally, the lungs (Figure 4) showed preserved alveolar structures, visible alveolar space, and an absence of inflammatory infiltrates (damage score = 0).

**TABLE 6:** Relative organ weight (mg/g) of female Swiss albino mice that received orally or intraperitoneally saline solution (negative control) or the coagulant *Moringa oleifera* lectin (cMoL) at 200 mg/kg.

Organs (mg/g)	NC (oral)	cMoL (oral)	*p*	NC (ip)	cMoL (ip)	*p*
Liver	61.0 ± 5.99	67.0 ± 4.89	0.6726	57.8 ± 3.06	67.0 ± 0.407	0.3135
Kidneys	11.3 ± 0.76	12.4 ± 0.39	0.7045	10.2 ± 1.13	12.3 ± 0.84	0.2227
Spleen	4.29 ± 0.26	6.29 ± 0.68	0.1424	4.52 ± 0.88	6.37 ± 0.73	0.1804
Heart	4.38 ± 0.71	4.50 ± 0.02	> 0.9999	4.12 ± 0.48	4.63 ± 0.56	> 0.9999
Lungs	6.33 ± 0.33	6.46 ± 0.74	> 0.9999	5.99 ± 0.22	7.11 ± 0.82	0.4199

*Note:* Values represented by mean ± SEM (*n* = 3/group). No significant differences (*p* > 0.05) were found between cMoL‐treated group and the respective control (oral or ip).

Abbreviation: NC: negative control.

### 3.3 Genotoxicity

Genotoxic evaluations coupled with acute toxicity studies are important to guarantee the safety of lectins. The assessment of genotoxicity is important because chemical, physical, or biological agents can interact with genetic material and cause mutations, which may be associated with genomic instability, cancer, malformations, metabolic disorders, and neurological diseases (Araldi et al. 2015). The comet assay is a sensitive and low‐cost technique that identifies single‐ and double‐strand breaks resulting from direct interactions with DNA and alkali–labile sites (W. Wang, Zhou, and Liu 2018). The genotoxicity assessment of cMoL through the comet assay (Table 7) did not demonstrate differences in the *DI* or *DF* values determined for animals treated with cMoL orally (*DI* = 20.67 ± 3.02; *DF* = 10.33 ± 1.73) or intraperitoneally (*DI* = 15.67 ± 2.23; *DF* = 9.83 ± 1.68), compared with the respective negative controls. cMoL promoted nucleoids damages classified mainly in low classes (0 and 1), whereas the positive control MTX (*DI* = 224.8 ± 21.33; *DF* = 86.0 ± 5.94) promoted nucleoid damages classified in high classes (2–4).

**TABLE 7:** Genotoxicity assessment by comet assay in female Swiss albino mice that received orally or intraperitoneally saline solution (negative control), the coagulant *Moringa oleifera* lectin (cMoL) at 200 mg/kg, or methotrexate at 20 mg/kg ip (positive control).

Treatment	Class					Damage index	Damage frequency
	0	1	2	3	4		
NC (oral)	89.0 ± 2.59^#^	7.0 ± 2.05^#^	2.17 ± 0.56^#^	1.17 ± 0.37^#^	0.67 ± 0.23^#^	17.5 ± 3.6^#^	11.0 ± 2.59^#^
*p* value_(PC)_	< 0.0001	0.0114	< 0.0001	< 0.0001	< 0.0001	< 0.0001	< 0.0001
cMoL (oral)	84.92 ± 1.91^#^	7.9 ± 1.30^#^	2.08 ± 0.51^#^	1.25 ± 0.41^#^	0.83 ± 0.35^#^	20.67 ± 3.02^#^	10.33 ± 1.73^#^
*p* value_(NC oral)_	0.8705	0.9983	> 0.9999	> 0.9999	> 0.9999	0.9992	0.9999
*p* value_(PC)_	< 0.0001	0.0356	< 0.0001	< 0.0001	< 0.0001	< 0.0001	< 0.0001
NC (ip)	86.42 ± 1.63^#^	11.0 ± 3.31	6.08 ± 0.81^#^	2.17 ± 0.44^#^	0.17 ± 0.11^#^	30.33 ± 3.61^#^	10.83 ± 1.71^#^
*p* value_(PC)_	< 0.0001	0.2421	< 0.0001	< 0.0001	< 0.0001	< 0.0001	< 0.0001
cMoL (ip)	89.75 ± 1.33^#^	6.67 ± 0.99^#^	1.92 ± 0.87^#^	1.5 ± 0.43^#^	0.17 ± 0.11^#^	15.67 ± 2.23^#^	9.83 ± 1.68^#^
*p* value_(NC ip)_	0.9336	0.5793	0.2347	0.9936	> 0.9999	0.7940	0.9993
*p* value_(PC)_	< 0.0001	0.0084	< 0.0001	< 0.0001	< 0.0001	< 0.0001	< 0.0001
PC	14.0 ± 5.94^$^ ^,^ *	17.4 ± 1.95*	23.27 ± 3.03^$^ ^,^ *	22.73 ± 2.61^$^ ^,^ *	23.36 ± 4.06^$^ ^,^ *	224.8 ± 21.33^$^ ^,^ *	86.0 ± 5.94^$^ ^,^ *

*Note:* Values represented by mean ± SEM (*n* = 5/group).

Abbreviations: NC: negative control; PC: positive control.

* *p* < 0.05 in relation to the NC (oral).

^$^ *p* < 0.05 in relation to the NC (ip).

^#^ *p* < 0.05 in relation to the PC.

**FIGURE 5:**
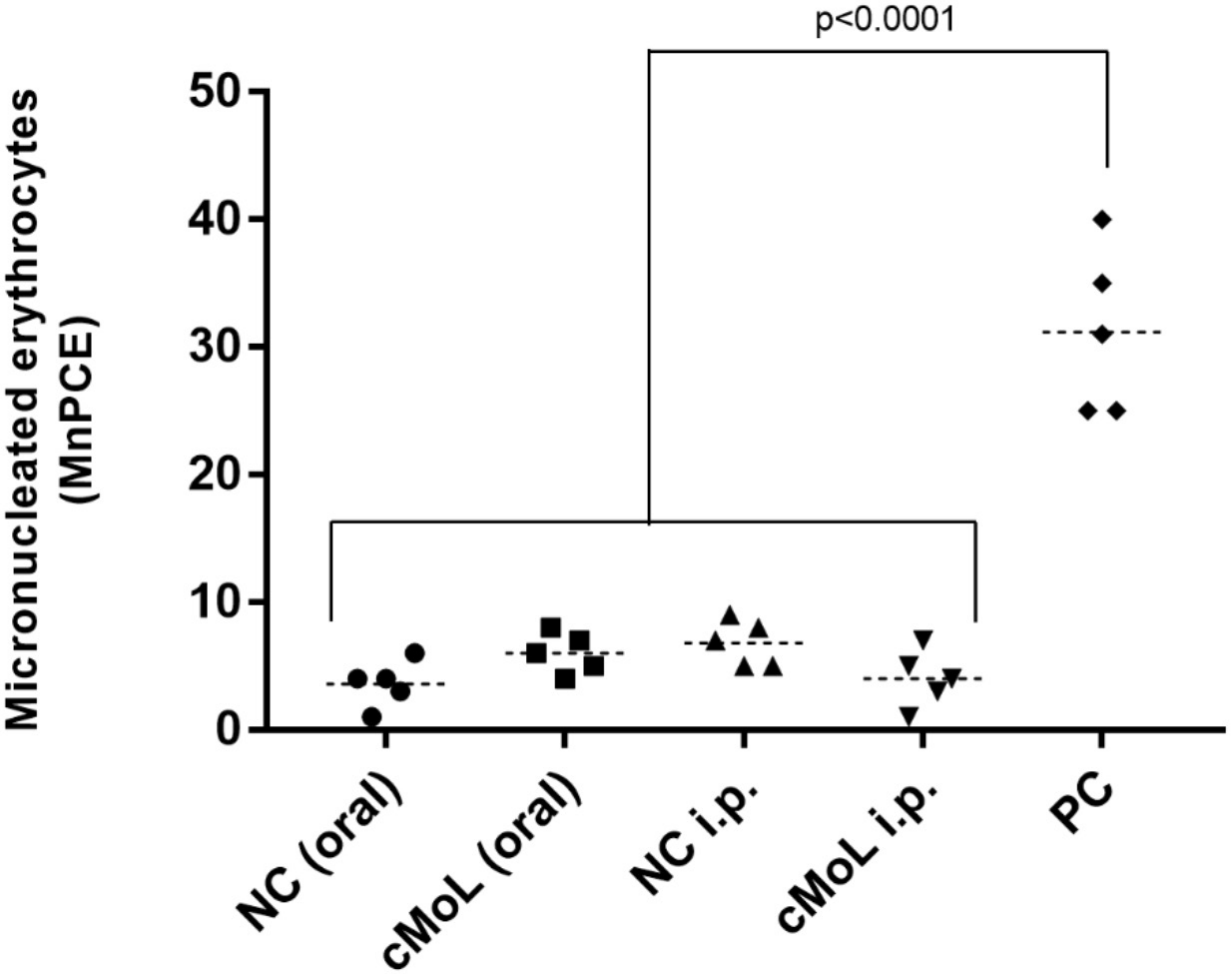
Genotoxicity assessment by micronucleus assay in female Swiss albino mice that received saline solution (negative control), cMoL at 200 mg/kg, or methotrexate at 20 mg/kg ip (positive control) orally or intraperitoneally.

The micronucleus test identifies the presence of acentric fragments expelled from the main nucleus during the final stages of anaphase. These fragments are formed via two mechanisms: chromosomal breaks (clastogenesis) or rupture of the mitotic apparatus (aneugenesis) (Araldi et al. 2015). The micronucleus test (Figure 5) indicated that oral (MnPCE = 6.0 ± 0.70) and intraperitoneal (MnPCE = 4.0 ± 1.00) treatments with cMoL did not significantly induce the formation of MnPCE. The negative controls presented MnPCE values of 3.6 ± 0.81 (oral treatment) and 6.8 ± 0.8 (intraperitoneal treatment). The positive control, MTX, significantly induced micronucleus formation (MnPCE = 31.2 ± 2.91). The genotoxic effect observed for MTX occurred because MTX is an antimetabolite that affects the synthesis of nucleotides and impairs DNA replication and cell growth (W. Wang, Zhou, and Liu 2018).

Some lectins have been suggested to induce genotoxicity by promoting DNA strand breaks generated by reactive oxygen species (ROS) (Bertholdo‐Vargas et al. 2009). In addition, the DNA damage caused by a mannose/glucose‐binding lectin from *Canavalia ensiformis* seeds (Concanavalin A) in the *Drosophila melanogaster* model is related to protein–carbohydrate interactions between the lectin and the plasma membrane, which activate mechanisms that induce oxidative stress and, consequently, DNA damage (A. M. D. O. Santos et al. 2022). The results obtained in this study corroborated previous findings that showed the absence of cMoL genotoxicity in lymphocytes in vitro evaluated through the comet assay (Barros et al. 2021).

## 4 Conclusion

The present study revealed that cMoL is a natural fungistatic agent against the fungi that cause cryptococcosis ( *C. neoformans* and *C. gattii*). cMoL promoted cell necrosis in all tested isolates and showed a synergistic effect with fluconazole against *C. neoformans* H99, thereby increasing the effect of the antibiotic. In addition to its antifungal activity, cMoL did not cause acute oral or intraperitoneal toxicity or genotoxicity in mice. These findings highlight the need for future studies to explore the biotechnological potential of cMoL as a low‐toxicity antifungal agent and evaluate the lectin effect in an in vivo infection model against *Cryptococcus*.

## Author Contributions

**Matheus Cavalcanti de Barros:** investigation, methodology, data curation, formal analysis, conceptualization, writing – original draft. **Sávia Soraia Santana da Silva:** investigation, methodology. **Alícia Natalie Silva dos Santos:** investigation, methodology. **Leydianne Leite de Siqueira Patriota:** investigation, methodology. **Gustavo Ramos Salles Ferreira:** investigation, methodology. **Pollyanna Michelle da Silva:** investigation, methodology. **Simone da Paz Leôncio Alves:** investigation, methodology. **Julliano Matheus de Lima Maux:** investigation, methodology. **Jacinto da Costa Silva Neto:** investigation, methodology. **Fabiana Aparecida Cavalcante Silva:** investigation, methodology. **Marilene Henning Vainstein:** methodology, funding acquisition, resources. **Luana Cassandra Breitenbach Barroso Coelho:** writing – review and editing. **Thâmarah de Albuquerque Lima:** writing – review and editing. **Thiago Henrique Napoleão:** funding acquisition, resources, supervision, writing – review and editing. **Patrícia Maria Guedes Paiva:** funding acquisition, resources, supervision, writing – review and editing.

## Conflicts of Interest

The authors declare no conflicts of interest.

## Data Availability

All data generated or analyzed during this study are included in this published article.
